# Bilateral renal artery embolism following trauma: A case report and systematic review

**DOI:** 10.1097/MD.0000000000045723

**Published:** 2025-11-07

**Authors:** Jintao Tang, Li He, Yong-An Xu, Qinqin Zhang, Shijia Chao, Yujun Liu

**Affiliations:** a Department of Thyroid and Breast Surgery, Nanxishan Hospital of Guangxi Zhuang Autonomous Region, Guilin, China; b School of Clinical Medicine, Guilin Medical University, Guilin, China; c Department of Emergency Medicine, Nanxishan Hospital of Guangxi Zhuang Autonomous Region, Guilin, China; d Department of Emergency Medicine, The Second Affiliated Hospital Zhejiang University School of Medicine, Hangzhou, China; e Department of Hospital Office, Guangxi Orthopaedic Hospital, Nanning, China.

**Keywords:** abdominal injury, case report, emergency, renal artery embolism, trauma

## Abstract

**Rationale:**

Post-traumatic renal artery embolism (RAE) represents a rare vascular emergency with diagnostic challenges due to its nonspecific presentation. This study aims to enhance clinical recognition through a comprehensive analysis of a bilateral RAE case and contemporary management strategies.

**Patient concerns:**

A 23-year-old male was admitted to the Emergency Department of the Second Affiliated Hospital, Zhejiang University School of Medicine, with acute chest and back pain and disturbance of consciousness following blunt abdominal trauma.

**Diagnoses:**

The diagnostic workup included contrast-enhanced computed tomography angiography and serum biomarkers.

**Interventions:**

Upon admission to the Emergency Intensive Care Unit, the patient underwent immediate continuous renal replacement therapy, vasoactive drugs, fluid replacement, alkalization of urine, and symptomatic treatment. After the patient’s condition improved, the spinal surgery performed thoracic vertebra reduction and internal fixation surgery.

**Outcomes:**

Serial renal function monitoring demonstrated complete functional recovery.

**Lessons:**

In the evaluation of post-traumatic abdominal pain, RAE should be systematically considered in the differential diagnosis following exclusion of acute surgical abdomen.

## 1. Introduction

Renal artery embolism (RAE), defined as occlusion of the renal artery or its branches leading to renal ischemia and parenchymal necrosis, represents a rare vascular emergency. The diagnostic conundrum stems from its nonspecific presentation (fewer than 30% of patients exhibit the classic triad of flank pain, hematuria, and hypertension) often delaying definitive diagnosis beyond the critical 12-hour therapeutic window. We report here a case of bilateral renal artery embolism secondary to abdominal trauma treated in the Emergency Department of The Second Affiliated Hospital of Zhejiang University School of Medicine. Based on a literature review, we aim to enhance clinicians’ understanding and management of this rare but critical condition.

## 2. Case report

A 23-year-old male was admitted to the Emergency Department with a 2-day history of chest pain and bilateral lower limb paralysis following trauma. The patient had a history of excessive alcohol consumption prior to the incident. He was found unconscious in a drainage ditch and transported to a local hospital via ambulance. The initial diagnosis from the local hospital was a closed abdominal injury. After 7 hours of fluid resuscitation and supportive care, the patient regained consciousness but complained of severe left thoracodorsal pain and exhibited complete loss of motor function in both lower extremities and retrograde amnesia regarding the traumatic event was noted. Notably, he denied chest tightness, dyspnea, nausea, vomiting, or headache. Following consciousness recovery, the patient developed progressive hypotension (nadir: 82/32 mm Hg) refractory to aggressive interventions at the local hospital, including massive fluid resuscitation (crystalloids/colloids), transfusion of 2 units of suspended red blood cells, and combined vasoactive agents (specific agents not documented) for shock management. He was subsequently transferred to our hospital for advanced care.

**Figure 1.**
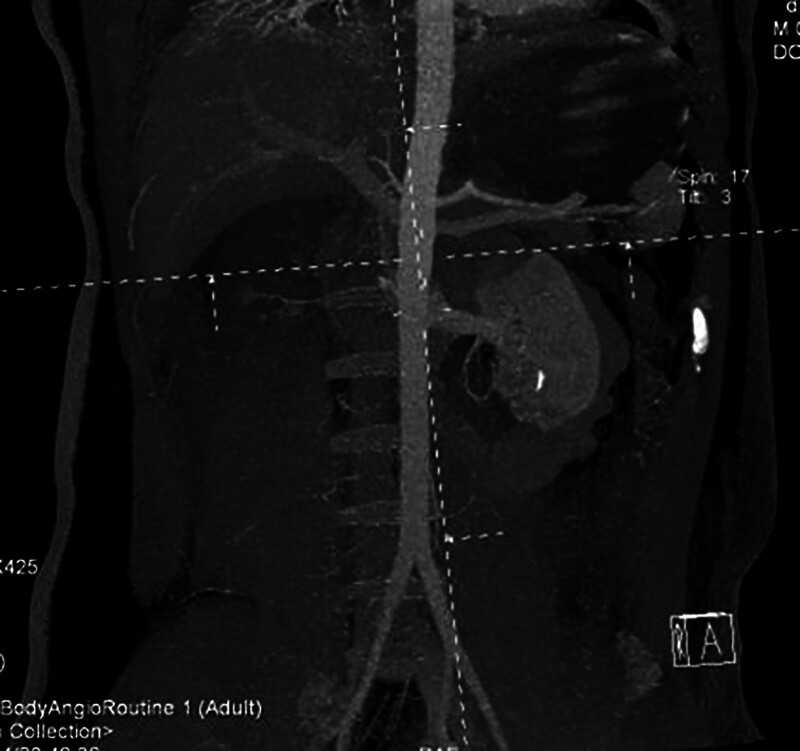
CTA demonstrates poor visualization of the right renal artery.

**Figure 2.**
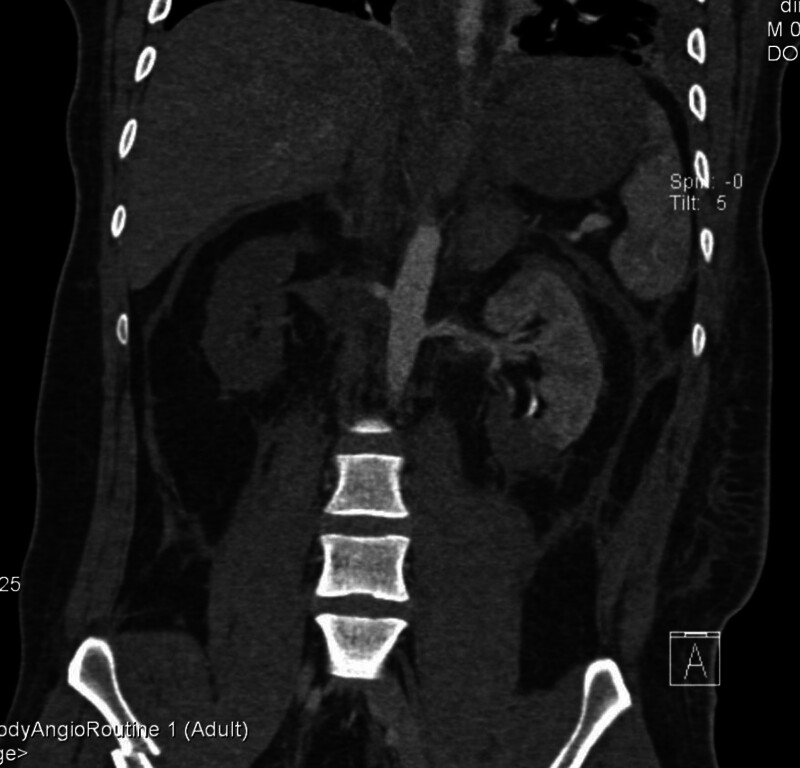
CTA demonstrates poor visualization of the right kidney with focal ischemia in the lower pole of the left kidney.

The patient had no significant medical history, denied history of hypertension, cardiovascular disease, or diabetes mellitus. There was no history of major trauma or surgery. On admission, the physical examination showed: temperature: 38.4℃, pulse: 118 beats/min, respiratory rate: 27 breaths/min, blood pressure: 146/82 mm Hg. The patient was conscious, responsive and coherent. There was no tenderness in the neck. The patient could move normally. There was no deformity in the chest. The compression sign was positive. The lungs had clear breath sounds. No obvious dry or wet rales were heard. The abdomen was soft. There was no tenderness or rebound pain. The pelvic compression and separation test was positive. There was obvious tenderness in the thoracic and lumbar vertebrae. Below the inguinal plane, sensation was lost. The muscle strength of both upper limbs was grade 5, while that of both lower limbs was grade 0. On the day of admission, the auxiliary examinations showed: serum creatinine: 156 μmol/L; blood urea nitrogen (BUN): 7.47 mmol/L; creatine kinase: 10744 U/L; lactate dehydrogenase (LDH): 984 U/L. B-ultrasound showed: no obvious abnormalities in the liver, gallbladder, pancreas, spleen, or both kidneys; a small amount of ascites; a small amount of effusion in the left thoracic cavity. Electrocardiogram: sinus tachycardia. After completing the computed tomography scan, the initial trauma assessment revealed bilateral parietal bone depressed fractures, bilateral lung contusions, multiple rib fractures on the left side combined with pneumothorax, hemiplegia due to fracture of the 12th thoracic vertebra, fractures of the left pubic symphysis and upper and lower branches, and contusions of both kidneys. Abdominal CTA showed poor visualization of the right renal artery and right kidney, and local ischemia in the lower pole of the left kidney (Figs. 1 and 2).

**Figure 3.**
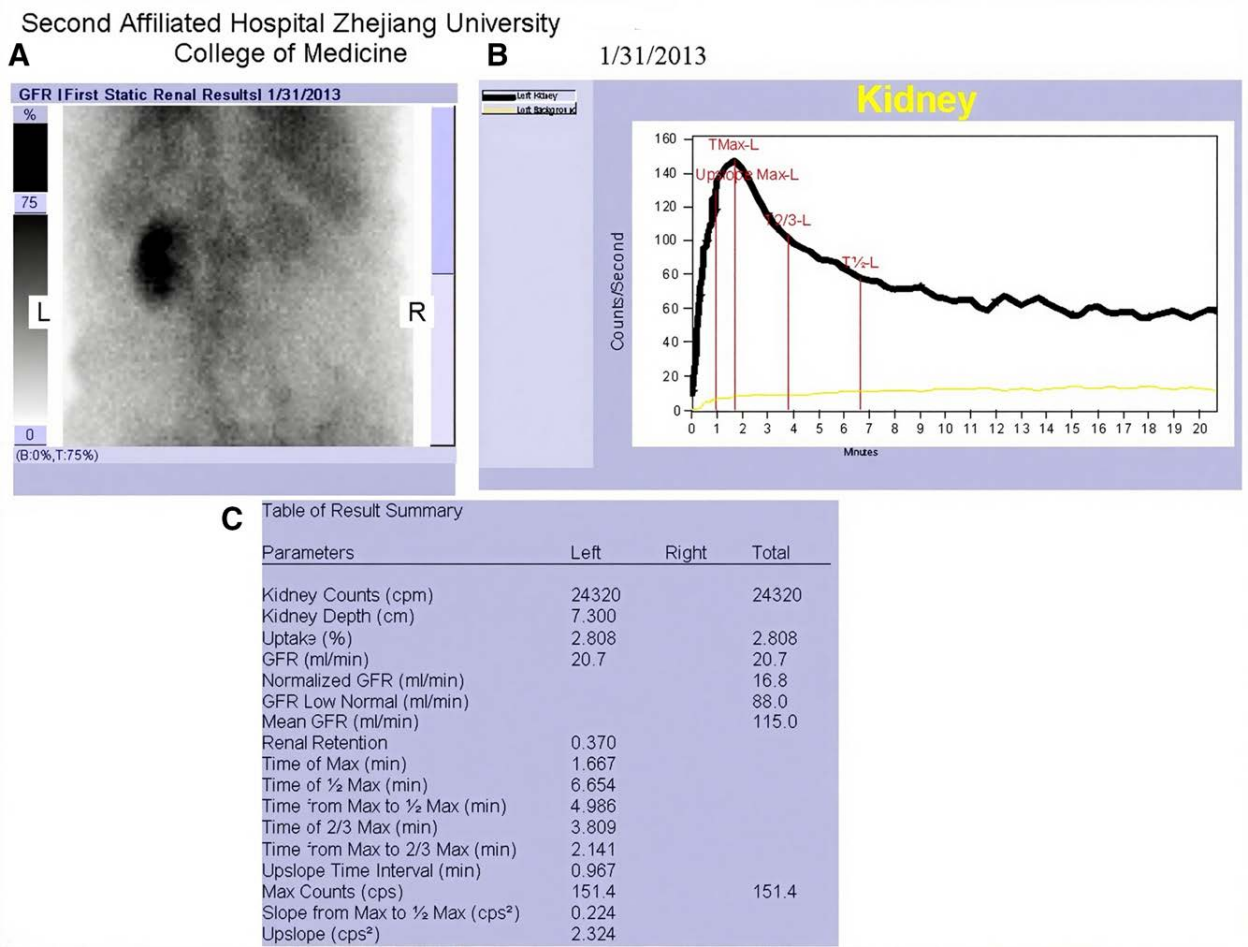
Renal dynamic imaging demonstrates impaired perfusion in the left kidney with moderately reduced glomerular filtration rate, while the right kidney exhibits no functional activity. (A) Renal blood perfusion imaging; (B) dynamic imaging of renal parenchymal function; (C) renal dynamic imaging summary.

After a multidisciplinary consultation and discussion in our hospital, based on the patient’s medical history, physical examination, and CTA findings, the diagnosis of bilateral renal artery embolism was confirmed.^[1]^ Given the severity of the condition and the high risk of thrombolysis, after communicating the situation with the physical examination, and CTA findings, conservative treatment was chosen. The specific treatment process was as follows: the patient was admitted to the emergency intensive care unit immediately and received active fluid replacement, analgesia, and alkalization of urine treatment. After 6 hours, the total urine output was 400 mL Repeated tests showed that creatine kinase and BUN had increased compared to before. Continuous renal replacement therapy (CRRT) was immediately initiated. After 12 hours, vasoactive drugs were discontinued, and after 48 hours of treatment, the urine output reached 2 mL/kg/h. CRRT was then stopped. BUN and creatinine were rechecked, and the renal dynamic imaging, the blood flow perfusion of the left kidney was impaired, and the glomerular filtration rate was moderately reduced; the right kidney was nonfunctional (Fig. 3). The patient’s condition improved, and after 7 days of treatment in the emergency intensive care unit, he was transferred to the general ward. On the 11th day, he was sent to the operating room under general anesthesia for posterior thoracic T12 reduction and internal fixation surgery. The operation went smoothly. On the 23rd day, the patient’s condition stabilized and was transferred to the rehabilitation ward. Since then, the patient has recovered well.

## 3. Discussion

RAE constitutes an acute vascular occlusion event characterized by thrombotic or embolic obstruction of renal arteries/branches, precipitating renal tissue ischemia and infarction.^[2]^ Current epidemiological data reveal that renal artery injury occurs in approximately 0.08% of closed abdominal trauma cases, with delayed intervention potentially progressing to secondary hypertension (23–41% incidence), end-organ failure (18% dialysis dependence), and necessitating nephrectomy in 9% to 15% of cases.^[3–5]^ A analysis by Cass et al^[6]^ of 1522 traumatic renal injuries demonstrated vascular involvement in 2.7% of cases, with unilateral RAE occurring in 1.2% and zero bilateral presentations. Notably, 80% to 100% of traumatic RAE cases present with multisystem trauma, predominantly abdominal injuries (67–85% comorbidity), with mechanistic patterns dominated by motor vehicle collisions (81%) versus falls (5%).^[7]^ When suffering from severe abdominal trauma, the abdominal wall and spine are simultaneously pulled and compressed, resulting in damage to the kidneys and renal arteries. The extensibility of the vascular intima is small, which easily leads to subintimal hemorrhage or gradual formation of thrombus. The compression of retroperitoneal hematoma is also one of the reasons.^[8]^ Relevant literature reports that traumatic RAE is more common on the left side, accounting for approximately 70%. The reason for this might be that the left renal artery is shorter and forms an angle with the aorta more slightly.^[9]^ In this case, the patient suffered severe trauma resulting in multiple fractures, pneumothorax and paraplegia. When high-energy injury occurs, the rightward displacement of the fracture ends of the thoracic vertebrae compressing the renal artery might be the cause of right renal embolism. The clinical manifestations of RAE lack specificity and may include low back pain, hematuria, proteinuria, and other nonspecific symptoms. The severity of these symptoms primarily depends on the extent and degree of renal ischemic necrosis. In clinical practice, RAE is often mistaken for urinary calculi.^[10]^ Studies have indicated that high LDH levels serve as a potential screening indicator for embolism, with LDH typically elevated in cases of renal infarction.^[11]^ High LDH can be used to predict acute renal artery embolism and may indicate an impending event. Diagnosis of RAE primarily relies on imaging examinations. Renal angiography remains the “gold standard” for diagnosing RAE; however, due to its invasive nature and significant radiation exposure, it is not typically the first-line diagnostic method.^[12,13]^ Ultrasound offers advantages such as rapidity, directness, and non-invasiveness, making it commonly used for follow-up after trauma-related abdominal effusion or thrombectomy. However, ultrasound is less reliable for diagnosing renal vascular lesions following abdominal trauma.^[14]^

In this case, the initial abdominal ultrasound failed to detect the lesion, likely due to the examiner’s experience and the lack of a vascular ultrasound probe. Early diagnosis of RAE often relies on CTA, which can not only reveal renal parenchymal lacerations, retroperitoneal hematomas, urine extravasation, and associated injuries to other organs, but also confirm the diagnosis through the presence of the cortical ring sign.^[15]^ Guidelines recommend that treatment for RAE should prioritize early vascular recanalization to preserve renal function as much as possible. Treatment options include anticoagulation, thrombolysis, and thrombectomy.^[16]^ Renal ischemia is generally tolerated for 60 to 90 minutes; timely revascularization can prevent further deterioration of renal function.^[17]^ Currently, the issue of the time window for treatment remains a subject of debate. Blum et al^[18]^ demonstrated that thrombolysis administered more than 180 minutes after disease onset does not improve patient outcomes. Conversely, some scholars argue that extending the thrombolysis time window may still allow for partial recovery of renal function in patients.^[19]^ Haas et al^[20]^ conducted a study involving 159 cases of renal artery embolism and found that the success rate of renal vascular recanalization following treatment was 56%. Patients who achieved vascular recanalization had an average ischemic time of less than 12 hours (range: 8–48 hours), whereas those who failed to achieve recanalization experienced an average ischemic time of 48 hours (range: 5–84 hours). Thus, further research is warranted to clarify this issue. With the ongoing advancements in interventional radiology, transarterial catheter-based interventions have increasingly replaced traditional open surgeries as the preferred treatment modality. These interventions include percutaneous transluminal renal angioplasty, renal artery stent placement, balloon catheter thrombectomy, and indwelling catheter contact thrombolysis.^[16]^ However, there remains a lack of randomized controlled trials comparing different interventional surgical methods for RAE, leading to ongoing controversy regarding method selection.^[11]^ Currently, no unified standard exists for the timing of CRRT initiation in patients with acute kidney injury. Most studies suggest that early CRRT initiation improves survival rates in acute kidney injury patients.^[21,22]^ In this case, given the presence of multiple systemic injuries, unstable condition, bleeding tendency, and a delay of two days from symptom onset to diagnosis, conservative treatment combined with early CRRT support was selected. After 1 week of treatment, renal function improved, and dynamic renal imaging revealed compensatory function in the left kidney.

## 4. Conclusions

In summary, traumatic renal artery embolism remains a rare yet critical clinical situation by severe sequelae, nonspecific presentation, and diagnostic challenges. This case suggests that for patients with abdominal trauma accompanied by unexplained pain, the possibility of renal artery embolism should be considered, and bilateral renal CTA should be completed as soon as possible to achieve early diagnosis. And targeted initiation of CRRT plays a crucial role in preventing further deterioration of renal function and improving prognosis.

## Author contributions

**Conceptualization:** Li He.

**Data curation:** Yong-an Xu, Shijia Chao.

**Supervision:** Yujun Liu.

**Writing – review & editing:** Jintao Tang, Qinqin Zhang.
